# Decreased Choroidal Vascular Index in Idiopathic Intracranial Hypertension

**DOI:** 10.1002/brb3.70258

**Published:** 2025-01-09

**Authors:** Hang Wang, Rui Wang, Le Cao, William Robert Kwapong, Ruishan Liu, Fayun Hu, Bo Wu

**Affiliations:** ^1^ Department of Neurology, West China Hospital Sichuan University Chengdu China

**Keywords:** choroidal vascular index, idiopathic intracranial hypertension, swept‐source optical coherence tomography

## Abstract

**Introduction:**

We aimed to explore the difference in choroidal vascular parameters using swept‐source optical coherence tomography (SS‐OCT) in patients with idiopathic intracranial hypertension (IIH) compared to controls. We also explored the ability of the choroidal parameters to reflect elevated intracranial pressure (ICP) in patients with IIH.

**Methods:**

This observational study recruited patients diagnosed with IIH and healthy controls. A lumbar puncture was performed for ICP measurement. All the participants underwent OCT examinations. The choroid was automatically segmented and imaged by the OCT tool. The parafoveal choroidal vascular volume (CVV) and choroidal vascular index (CVI) were calculated in 3 mm annulus and 6 mm annulus.

**Results:**

A total of 80 patients with IIH (34.67 ± 11.00 years; 37.50% males) and 92 controls (34.50 ± 12.08 years; 36.96% males) were included in the final analysis. Patients with IIH had higher BMI (< 0.001) and poor visual acuity (< 0.001) compared with controls. Patients with IIH demonstrated significantly lower parafovea CVI in both annuluses (*p* = 0.003 for 3 mm annulus, *p* = 0.001 for 6 mm annulus) compared to controls. Mean parafovea CVI in both annuluses was significantly correlated with ICP level (*p* = 0.014 for 3 mm annulus, *p* = 0.015 for 6 mm annulus). The combination of CVV and CVI in a 6 mm annulus demonstrated the highest diagnostic value with a mean AUC of 0.818.

**Conclusion:**

CVI may serve as a potential marker for identifying IIH and reflecting ICP changes.

## Graphical abstract

Choroidal vascular parameters, measured by SS‐OCT, differ significantly between patients with idiopathic intracranial hypertension (IIH) and controls. Patients with IIH show reduced parafoveal choroidal vascular index (CVI), correlated with intracranial pressure. The combination of choroidal vascular volume (CVV) and offers high diagnostic value for detecting IIH.

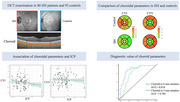

## 1 Introduction

Idiopathic intracranial hypertension (IIH) is a condition characterized by signs and symptoms of increased intracranial pressure (ICP) (Kwapong et al. 2023; Wang et al. 2023). There is a high risk of permanent vision loss associated with IIH. IIH can result in optic neuropathy associated with chronic or severe papilledema. Optic disc hemorrhages, papilledema, and optic nerve head atrophy are manifestations of IIH as previously reported (Wang et al. 2023; Acheson 2006). Recent studies (Kwapong et al. 2023; Nichani and Micieli 2021) have shown that retinal manifestations such as macula exudate and retinal folds are common in patients with IIH.

Optical coherence tomography (OCT)/OCT angiography (OCTA), a retinal imaging modality, has been used to detect retinal structural and microvascular changes in IIH. Prior reports (Kwapong et al. 2023; Kocer et al. 2023; Moreno‐Ajona, McHugh, and Hoffmann 2020) showed patients with IIH have thinner retinal structural thicknesses and reduced retinal microvascular densities compared to controls. These studies suggested that OCT/OCTA, a noninvasive imaging modality, has the potential to quantify neurodegeneration and microvascular vascular impairment associated with IIH; in addition, these studies demonstrated that retinal structural and microvascular changes are associated with their clinical consequences.

Choroidal changes, such as choroidal neovascular membranes and choroidal folds, have long been detailed in the eyes of IIH (Ariello et al. 2022). The swept‐source OCT (SS‐OCT) has a fast scanning speed, which allows for denser scan patterns and larger scan areas. Besides, it has a longer wavelength and a lower sensitivity roll‐off, resulting in improved light penetration through the retinal pigment epithelium (RPE) as well as deeper layers such as the choroid.

Since the choroid is underexplored, its potential as a surrogate marker for IIH remains inconclusive. Our study aimed to explore the choroidal vascular parameters measured using SS‐OCT in patients with IIH compared to controls. We also explored the ability of the choroidal parameters to reflect ICP changes in patients with IIH.

## 2 Materials and Methods

### 2.1 Participants

This observational study recruited patients who were diagnosed with IIH at the Neurology Department of West China Hospital, Sichuan University, according to international criteria (Friedman, Liu, and Digre 2013) from April 2021 to October 2023. Diagnostic criteria for IIH encompassed comprehensive neurological symptoms, neuroimaging (magnetic resonance imaging [MRI]/magnetic resonance venography [MRV]), lumbar puncture, and cerebrospinal fluid (CSF) analysis.

The exclusion criteria are as follows: (1) with the history of cerebral vascular disease, including stroke, carotid artery stenosis, arteriovenous malformation, and arteritis; (2) with history of other neurologic disease, including neuromyelitis optica spectrum disorders; (3) with medication for lowering ICP or immunosuppressant therapy; (4) with prior interventions to manage intracranial hypertension, including CSF shunting, venous sinus stenting and optic nerve sheath fenestration (ONSF). Age‐ and sex‐matched healthy controls were individuals undergoing routine health examinations, free from neurological or ophthalmological pathologies. Written informed consent was acquired from each participant. The research was approved by the Ethics Committee of West China Hospital, Sichuan University, China (No. 2020 [922]) and followed the tenets of the Declaration of Helsinki.

### 2.2 Clinical Features Collection

Demographic information (age, gender, body mass index [BMI]) and vascular risk factors, including hypertension, diabetes, dyslipidemia, smoking status, and drinking status, were recorded for all participants. ICP was measured by lumbar puncture at the L3–L4 intervertebral space, with pressure readings stabilized using a 500‐mm manometer.

### 2.3 Comprehensive Ophthalmic Examination

All the participants underwent anterior segment examination with slit lamp, intraocular pressure (IOP) measurement, and color fundus photography to identify and subsequently exclude individuals with significant ocular conditions such as advanced cataract, retinal vascular occlusion, retinal hemorrhages, diabetic retinopathy, and glaucoma.

The severity of papilledema in each eye was graded using the modified Frisen Scale, ranging from a score of 0–5 based on fundus photography. Non‐severe patients were defined as those with a modified Frisen score of less than 4 (Wang et al. 2023).

Best corrected visual acuity (VA) was measured with a standard logarithm VA chart and the results were converted to the logarithm of VA (log VA) format for analysis. Ophthalmic examination and OCT examination were performed on the same day of the lumbar puncture

### 2.4 Swept‐Source OCT Examination

OCT examination was conducted before any pharmacological treatment and surgical intervention was performed to lower ICP. OCT (SVision Imaging; version 2.1.016) was used to image the choroidal structure and vasculature of participants. The specifications of the OCT are well‐described in our previous studies (Kwapong et al. 2023; Wang et al. 2023; Cao et al. 2023).

The choroid was automatically segmented and imaged by the OCT tool, covering an area of 6 × 6 mm^2^ centered on the fovea. The choroidal vascular volume (CVV) was defined as the vascular volume from the basal border of the RPE–Bruch membrane complex to the choroidoscleral junction. Choroidal vascular index (CVI) was described as the ratio of the choroidal vascular luminal volume to the total choroidal volume.

The parafoveal area was defined as an annulus with an inner diameter of 1 mm and an outer diameter of 6 mm. We analyzed the mean parafoveal CVV and CVI values both in 3 mm annulus and 6 mm annulus. The two annuluses were divided into four sectors (superior [S], temporal [T], nasal [N], and inferior [I]) according to the ETDRS grid, and the CVI and CVV in each quadrant were calculated.

OCT images with a signal quality of below 7 were excluded from our analysis. OCT data presented adhered to the OSCAR‐IB quality criteria (Tewarie et al. 2012) and APOSTEL recommendation (Aytulun et al. 2021) in our study.

### 2.5 Data Analysis

Characteristics of participants were described as mean ± standard deviation (SD), median (interquartile range [IQR]) for continuous variables and frequencies with percentages for categorical variables. The Fisher's exact test, *t*‐test, or Kruskal–Wallis test were used to compare clinical characteristics between IIH and controls.

Generalized estimating equations (GEEs) were used to compare choroidal metrics between the two groups. The covariates were set as age, gender, BMI, and vascular risk factors and the working correlation matrix were set as exchangeable considering the correlation of two eyes. Multivariable linear regressions were used to investigate the association between choroidal metrics and modified Frisen scores or log VA based on each eye of patients with IIH; while GEEs were also conducted when exploring the association between choroidal metrics and ICP based on each patient with IIH. The covariates of linear regression were the same as GEEs. The added variable plot was used to show the partial correlation between clinical features and choroidal metrics. The heatmap was used to show a correlation between choroidal metrics in four quadrants and clinical features and the color represents *z* values in each regression.

When exploring the discriminative importance of distinguishing IIH for each choroidal metric, an univariable logistic regression model using each separate metric was performed and the area under the curve (AUC) from the receiver operating characteristic (ROC) was used to evaluate the models. Finally, we built two support vector machine (SVM) classifiers with a radial kernel to predict IIH using choroidal metrics in two annuluses (diameter of 3 and 6 mm), combined with age, gender, BMI, and vascular risk factors. The hyperparameters of the SVM classifiers were tuned using a grid‐search method; and the combination of gamma and parameter C, which maximized the mean prediction accuracy of the SVM classifier, was chosen. The gamma value and cost parameter C were tested for 0.001, 0.01, 0.1, 1.0, 10, and 100 and further refined to 1–10. Classifier performance (mean AUC) was evaluated on the test dataset using fivefold cross‐validation. The logistic regression model and SVM classifiers were also used to distinguish non‐severe patients with IIH from control.

All statistical analyses and plotting were conducted using R (version 4.23), and *p* < 0.05 was considered significant. “pROC” and “plotROC” packages were used for AUC analysis, while “e1071” was for SVM construction, “caret” for cross‐validation.

## 3 Results

### 3.1 Baseline Characteristics

**FIGURE 1:**
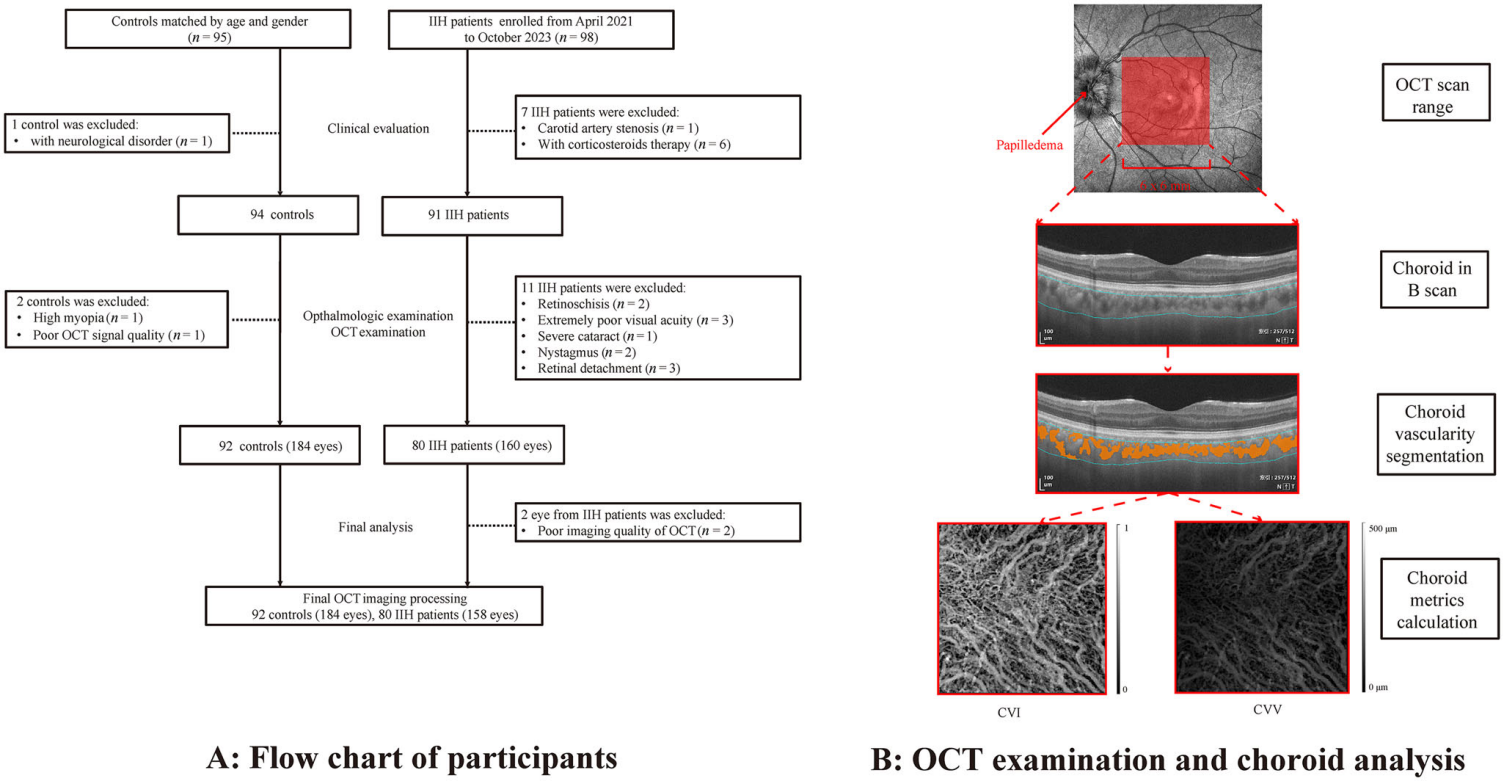
(A) The flowchart of participants in our study. (B) The choroid and choroid vascularity were segmented in the OCT tool in an area of 6 × 6 mm^2^ centered on the fovea; consequently, choroidal vascular index (CVI) and choroidal vascular volume (CVV) were calculated. IIH, idiopathic intracranial hypertension; OCT, optical coherence tomography.

A total of 98 patients with IIH and 95 controls were recruited in our study. Eighteen patients with IIH were excluded due to carotid artery stenosis or receiving corticosteroid therapy or ophthalmologic disorders (Figure 1). A total of 80 patients with IIH (34.67 ± 11.00 years; 37.50% males) and 92 controls (34.50 ± 12.08 years; 36.96% males) were included in the final data analysis. Patients with IIH had higher BMI (< 0.001) and poor VA (< 0.001) compared with controls, with a notable 89.87% of patients with IIH presenting with papilledema.

### 3.2 Comparison of CVI and CVV Between IIH and Controls

Patients with IIH demonstrated significantly lower parafovea CVI in both 3 mm annulus (*p* = 0.003) and 6 mm annulus (*p* = 0.001) compared to controls (Table 1). However, no significant difference in CVV was observed between IIH and controls in either annulus (Table 1).

**TABLE 1:** Characteristics of participants.

	Control	IIH	*p*
*n*	92	80	
Eyes, *n*	184	158	
Age, year	34.50 ± 12.08	34.67 ± 11.00	0.921
Gender, male	34 (36.96%)	30 (37.50%)	1
Smoking, *n*	9 (9.78%)	13 (16.25%)	0.299
Drinking, *n*	6 (6.52%)	10 (12.50%)	0.279
Diabetes, *n*	2 (2.17%)	4 (5.00%)	0.555
Hypertension, *n*	5 (5.49%)	9 (11.25%)	0.276
Dyslipidemia, *n*	4 (4.35%)	13 (16.25%)	0.019
VA, log VA	0.00 (0.00–0.00)	−0.10(−0.30 to 0.00)	< 0.001
BMI	23.51 (21.62–25.47)	26.04 (23.44–28.88)	< 0.001
ICP, mmH_2_O		300 (250–330)	
Disease duration, days		30 (14–60)	
Papilledema, *n*		142 (89.87%)	
Modified Frisen scores		3 (1–4)	
Choroid in 3 mm annulus			
CVI	0.44 ± 0.07	0.41 ± 0.07	0.003
CVV	0.83 ± 0.23	0.89 ± 0.25	0.081
Choroid in 6 mm annulus			
CVI	0.42 ± 0.06	0.40 ± 0.05	0.001
CVV	3.03 ± 0.84	3.29 ± 0.97	0.054

Abbreviations: BMI, body mass index; CVI, choroidal vascular index; CVV, choroidal vascular volume; ICP, intracranial pressure; IIH, idiopathic intracranial hypertension; log VA, logarithm of visual acuity; VA, visual acuity.

**FIGURE 2:**
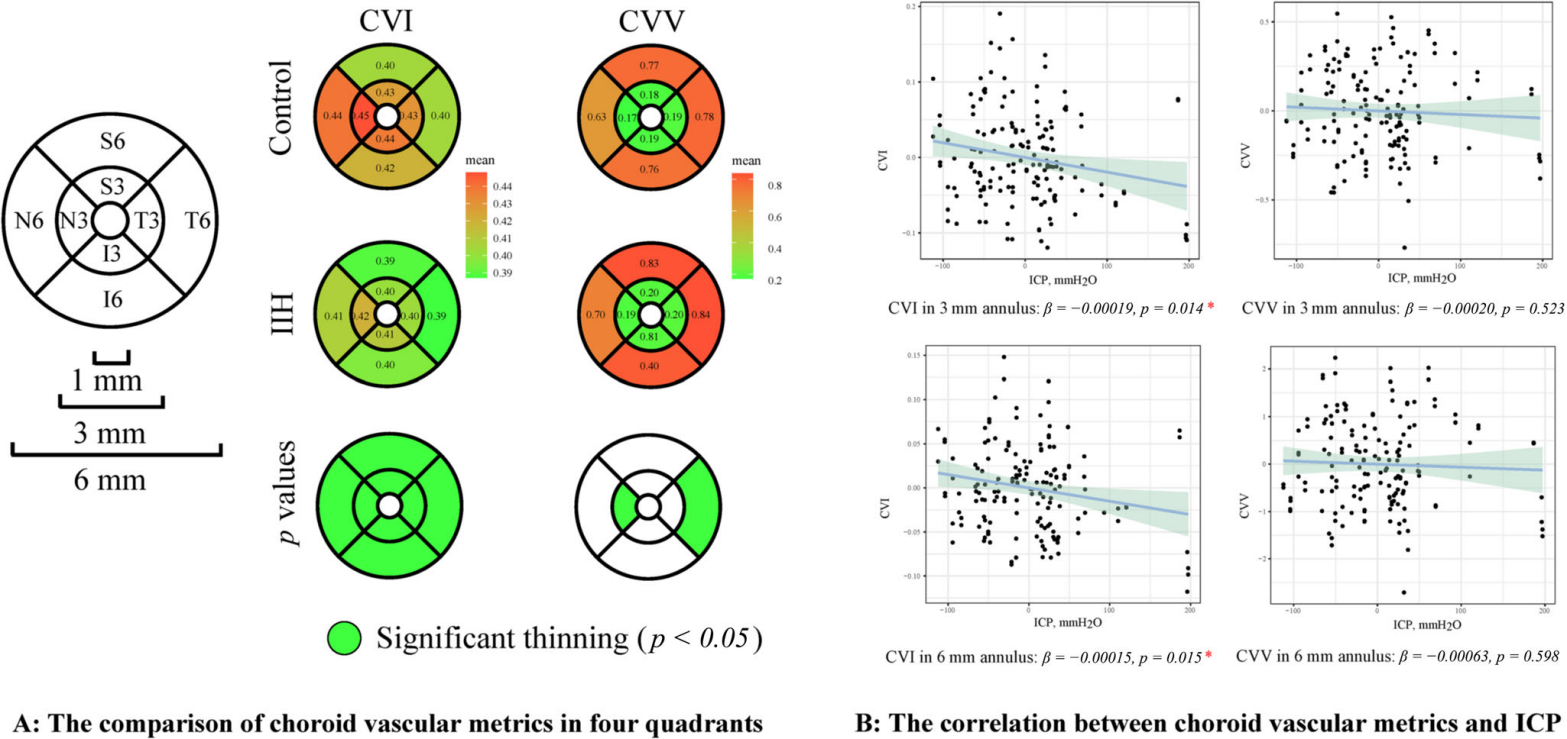
(A) The comparison of choroid vascular metrics in four quadrants; (B) the correlation between choroid vascular metrics and ICP. CVI, choroidal vascular index; CVV, choroidal vascular volume; ICP, intracranial pressure.

Further analysis of CVI and CVV across four quadrants (S, T, I, and N) in the two annuluses revealed significantly lower CVI in each quadrant for patients with IIH (all *p* < 0.05, Figure 2). Nonetheless, no significant differences in CVV were detected in quadrants analysis except in temporal quadrants of 6 mm annulus and nasal quadrants of 3 mm annulus. The comparison of CVI and CVV between IIH and controls in 3 and 6 mm annulus was shown in Figure 2.

### 3.3 Correlation Between Clinical Features and Choroid Parameters

Mean parafovea CVI in both annuluses was significantly correlated with ICP level (*p* = 0.014 for 3 mm annulus and *p* = 0.015 for 6 mm annulus), whereas no significant correlation between CVV and ICP was found (Figure 2). Moreover, no correlation was observed between CVI, CVV, and modified Frisen scores or log VA (Figure S1).

Figure S2 illustrates the correlation between clinical features and choroidal parameters in each quadrant (S, T, I, and N), highlighting that CVI quadrantal parameters correlated with ICP, except for CVI in nasal quadrants.

### 3.4 Diagnostic Value of Choroid Parameters

**FIGURE 3:**
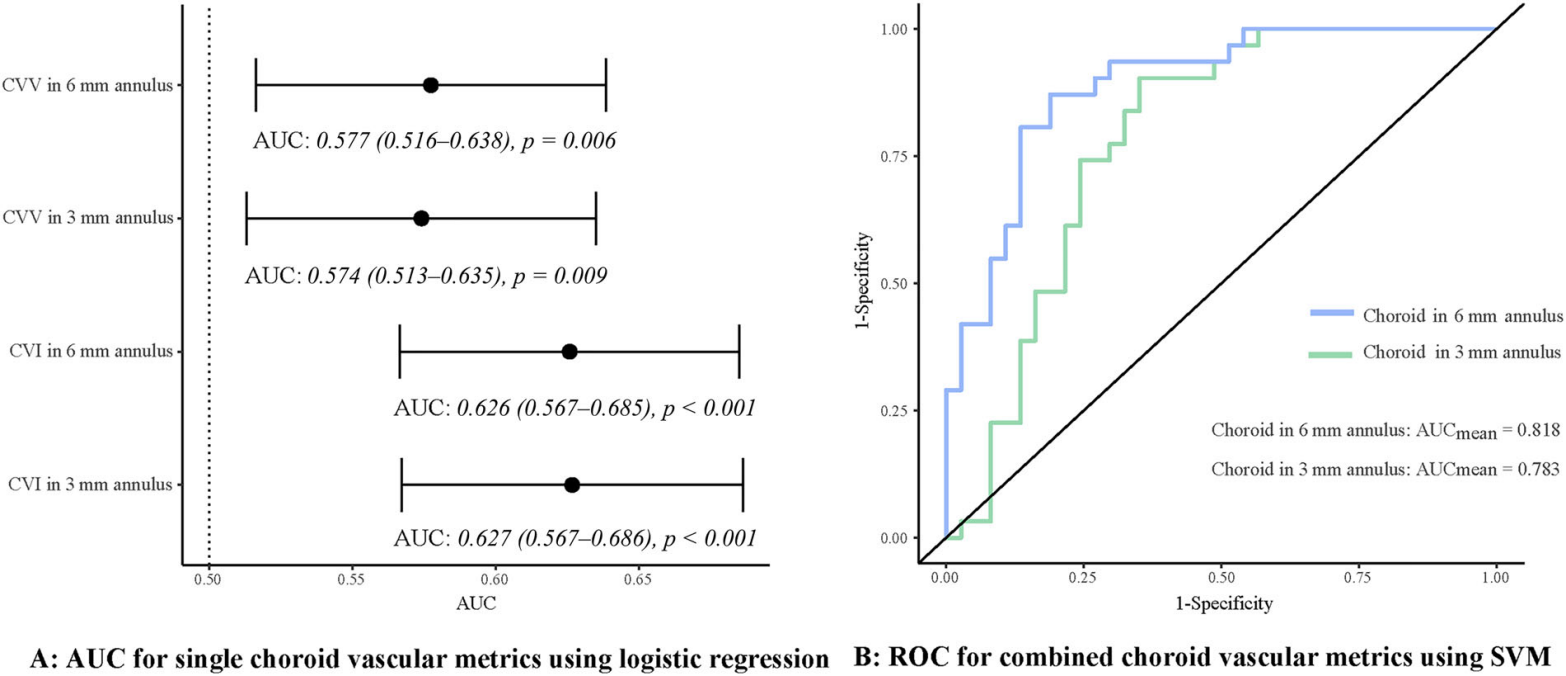
(A) AUC for single choroid vascular metrics using logistic regression; (B) ROC for combined choroid vascular metrics using SVM. AUC, area under the curve; CVI, choroidal vascular index; CVV, choroidal vascular volume; ROC, receiver operating characteristic; SVM, support vector machine.

Using the logistic regression model (Figure 3A) to discriminate IIH from controls, the AUC of CVV in 3 mm annulus was 0.574 (0.513–0.635), and that in 6 mm annulus was 0.577(0.516–0.638). Notably, the CVI exhibited a higher diagnostic value with an AUC of 0.626 (0.567–0.685) in the 6 mm annulus and 0.627 (0.567–0.686) in the 3 mm annulus. In addition, the SVM model (Figure 3B) combining CVV and CVI, indicated the comprehensive choroid parameters in 3 mm annulus had a mean AUC of 0.783, while those in 6 mm annulus demonstrated the highest diagnostic value with a mean AUC of 0.818. Furthermore, to distinguish non‐severe patients with IIH from the control, the combination of CVV and CVI demonstrated a mean AUC of 0.761 in 3 mm annulus and 0.788 in 6 mm annulus, as shown in Figure S3.

## 4 Discussion

We used SS‐OCT imaging and automated algorithms to assess the choroidal changes in patients with IIH. In addition to providing deeper penetration of light and better visualization of the choroid, SS‐OCT imaging also facilitates the use of automated choroidal parameters. A dense, volumetric choroidal measurement can provide a better, more robust analysis of choroidal changes associated with IIH. Here, we showed that patients with IIH had lower CVI compared to controls. We also showed that in patients with IIH, lower CVI was associated with higher ICP. Choroidal parameters from SS‐OCT images may serve as a potential marker for detecting elevated ICP in IIH.

Choroidal abnormalities in patients with IIH do not only involve structural changes such as choroidal folds and wrinkles as previously reported (Ariello et al. 2022) but also involve choroidal hypoxia, in chronic cases, and neovascularization may occur. Here, we found higher CVV and lower CVI in patients with IIH compared to controls; however, the difference in CVV between patients with IIH and control was marginally positive, which may be attributed to the relatively small sample size. Over 85% of the circulation in the eye comes from the choroidal circulation (Nickla and Wallman 2010); CVV represents the choroidal vascular volume, while CVI describes the density of the choroidal vascular network. Reports also suggest that optic nerve abnormalities in patients with IIH result in choroidal abnormalities that are suggested to be linked with choroidal hypoxia (Ariello et al. 2022; Duchnowski and Rodman 2010). Here, we suggest that higher CVV may be due to the changes in blood flow in the ciliary arteries and the occurrence of choroidal neovascularization during the course of IIH. In addition, accumulating studies have reported the congestion of the veno‐glymphatic system of the brain may result in decreased clearance of interstitial fluid and subsequent CSF accumulation, contributing to the development of IIH (Licastro et al. 2024; Lenck et al. 2018). The choroid comprises vessels and stroma, which are supplied by posterior ciliary arteries and drained by vortex veins. The congestion of the veno‐glymphatic system and elevated intracranial hypertension impairs vortex vein drainage, subsequently leading to an increase in choroidal volume (Ozdemir and Çevik 2020). Disproportionate changes in CVV and stromal volume result in a reduction in CVI.

Recent studies suggest that increased ICP results in choroidal changes; choroidal folds, a clinical manifestation commanly seen on fundus images and OCT in severe patients with IIH. Previous studies have tried to explore the association between choroidal parameters and ICP. A seminal population‐based study demonstrated that choroidal thickness was correlated with ICP but this article was based on a major assumption that ICP can be assessed by a formula combining diastolic blood pressure, age, and BMI (Jonas et al. 2014). Our study indicated that increased ICP was correlated with lower CVI. The elevated ICP increases cerebral venous pressure and hinders vortex vein drainage, subsequently leading to an increase in choroidal stroma, resulting in a reduction of CVI (Ozdemir and Çevik 2020). Notably, CVI is a sensitive marker, reflective of changes in both choroidal vasculature and stroma, while remaining relatively unaffected by age, IOP, and axial length (Abdolrahimzadeh et al. 2023). Therefore, CVI may serve as a marker for elucidating choroidal vascular disorder and reflecting ICP changes in patients with IIH.

According to the ROC curve analysis, CVI had a higher discriminating power than CVV in detecting choroidal changes in patients with IIH compared to controls. A combination of both choroidal measurements yielded even greater detection capability. Our research indicates that OCT‐derived choroidal metrics are valuable for detecting the choroidal changes associated with IIH, highlighting the noninvasive advantage of OCT imaging. Our previous reports (Kwapong et al. 2023; Wang et al. 2023) showed that the OCT is sensitive to structural and microvascular changes in the optic nerve head and retina of patients with IIH. Identifying these choroidal changes before the occurrence of apparent choroidal signs, such as choroidal neovascular membranes and choroidal folds, may help clinicians implement early treatment and may also be useful in predicting the progression of structural and microvascular complications associated with IIH.

Potential limitations of our study should be mentioned. First, this is a cross‐sectional observational study, and thus, cause‐and‐effect interpretation of CVI and ICP is limited. Second, the lack of visual field assessments leaves the relationship between visual field changes and OCT features unexplored, necessitating further investigation. Finally, CVI changes may not be unique to IIH but could also occur in other conditions associated with elevated ICP, such as intracranial mass lesions or cerebral hemorrhage. Further studies encompassing a broader spectrum of diseases are needed to validate and extend our findings.

## 5 Conclusion

We demonstrated that patients with IIH had lower CVI compared with controls and CVI had promising diagnostic value in differentiatinIIH from controls. Importantly, we showed that increased ICP was correlated with decreased CVI in patients with IIH. This suggests CVI may serve as a potential marker for reflecting ICP changes. The assessment of choroidal features could provide valuable insights into the pathophysiology of IIH.

## Author Contributions

**Hang Wang**: conceptualization, methodology, software, data curation, investigation, writing–original draft, formal analysis. **Rui Wang**: data curation, methodology, investigation, formal analysis, writing–original draft. **Le Cao**: conceptualization, investigation, software, formal analysis, data curation, visualization. **William Robert Kwapong**: writing–review and editing, conceptualization, methodology, investigation. **Ruishan Liu**: formal analysis, data curation. **Fayun Hu**: writing–review and editing. **Bo Wu**: writing–review and editing, supervision, project administration.

## Conflicts of Interest

The authors declare no conflicts of interest.

### Peer Review

The peer review history for this article is available at https://publons.com/publon/10.1002/brb3.70258

## Ethics Statement

The West China Hospital of Sichuan University Ethics Committee approved the study (Ethics number 2020[922]).

## Supporting information



Supplementary Figure 1: The correlation of choroid vascular metrics, modified Frisen score, and visual acuity. CVI, choroidal vascular index; CVV, choroidal vascular volume; MFS, modified Frisen scores; VA, visual acuity

Supplementary Figure 2: The correlation of choroid vascular metrics in four quadrants and clinical features (intracranial pressure, modified Frisen scores, visual acuity). CVI, choroidal vascular index; CVV, choroidal vascular volume; S, superior; T, temporal; N, nasal; I, inferior; ICP, intracranial pressure; MFS, modified Frisen scores; VA, visual acuity;

Supplementary Figure 3: (A) AUC for single choroid vascular metrics using logistic regression in control and IIH patient of non‐severe stage; (B) ROC for combined choroid vascular metrics using SVM in control and IIH patient of non‐severe stage. CVI, choroidal vascular index; CVV, choroidal vascular volume; AUC, area under the curve; IIH: idiopathic intracranial hypertension; ROC, receiver operating characteristic; SVM, support vector machine.

## Data Availability

The data that support the findings of this study are available on request from the corresponding author upon reasonable request.
