# Supplementary figures and images for: Decreased Choroidal Vascular Index in Idiopathic Intracranial Hypertension
Source: Brain Behav. 2025 Jan 9;15(1):e70258. doi: 10.1002/brb3.70258 (PMC11726707; doi:10.1002/brb3.70258)

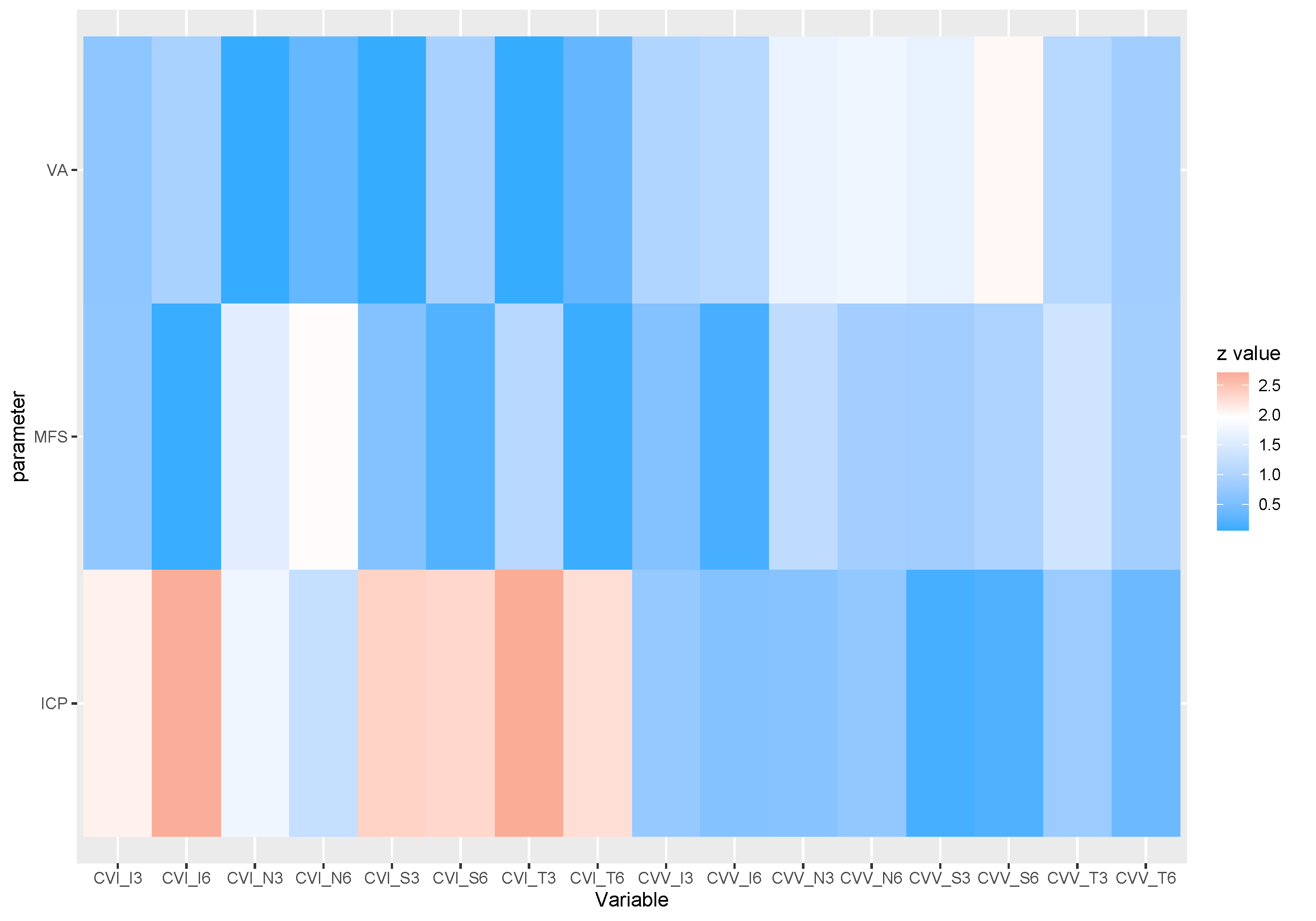

Supplement: Supplementary file 2 — Supplementary Figure 2: The correlation of choroid vascular metrics in four quadrants and clinical features (intracranial pressure, modified Frisen scores, visual acuity). CVI, choroidal vascular index; CVV, choroidal vascular volume; S, superior; T, temporal; N, nasal; I, inferior; ICP, intracranial pressure; MFS, modified Frisen scores; VA, visual acuity; [file BRB3-15-e70258-s003.tiff]
